# New record of strawberry leopard (*Panthera pardus*) in Selous Game Reserve, Tanzania

**DOI:** 10.1002/ece3.11542

**Published:** 2024-07-08

**Authors:** Charlotte E. Searle, Paolo Strampelli, Singira N. Parsais, Leonard J. Haule, Kandey K. Olesyapa, Nasri D. Salum, Germanus Hape, Manase Elisa, Daniel Mathayo, Joseph Kaduma, Neema Malulu, Nyasatu Mkaka, Justine Robert, Dennis Ikanda, Samuel Mtoka, Kathryn Doody, Alex L. Lobora, Amy J. Dickman

**Affiliations:** ^1^ Wildlife Conservation Research Unit (WildCRU), Department of Biology University of Oxford Tubney UK; ^2^ Lion Landscapes Iringa Tanzania; ^3^ Panthera New York New York USA; ^4^ Tanzania Wildlife Management Authority (TAWA) Morogoro Tanzania; ^5^ Tanzania Wildlife Research Institute (TAWIRI) Arusha Tanzania; ^6^ Tanzania National Parks (TANAPA) Arusha Tanzania; ^7^ Frankfurt Zoological Society (FZS) Frankfurt Germany

**Keywords:** African leopard, colour morph, Miombo woodland, Nyerere‐Selous ecosystem, *Panthera pardus*, phenotype

## Abstract

Strawberry or red leopards are a rare colour morph of leopard (*Panthera pardus*) characterised by spot markings that are red or brown instead of black, thought to be a result of a mutation in the tyrosinase‐related protein (TYRP1) gene. We report the first record of this phenotype on the African continent outside of South Africa, from Selous Game Reserve in southern Tanzania. One female leopard with strawberry colouration was documented out of 373 individual leopards (0.3%) identified through camera trap surveys conducted from 2020 to 2022 over a combined area of more than 4600 km^2^ in the Nyerere‐Selous landscape.

## Graphical abstract

Strawberry leopards are a rare colour morph of leopard (*Panthera pardus*) characterised by spot markings that are red or brown instead of black. We report the first record of this phenotype for the African continent outside of South Africa, from Selous Game Reserve in southern Tanzania. One female leopard with strawberry colouration was documented out of 373 individual leopards (0.3%) identified through camera trap surveys conducted in the Nyerere‐Selous ecosystem from 2020 to 2022.

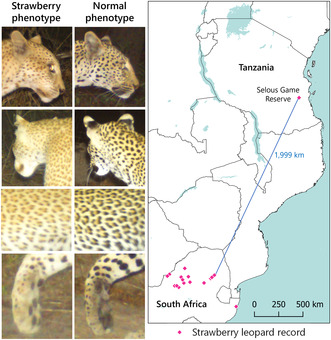

## 1 INTRODUCTION

Strawberry leopards – also referred to as red, pink, or golden leopards – are a rare colour morph of leopard (*Panthera pardus*) characterised by spot markings that are red or brown instead of black, blue eyes, and pink skin. It was previously thought that this colouration was a result of erythrism, a genetic mutation that causes an absence of a normal dark pigment or excessive production of red pigment (Dell'Amore, 2012; Pirie et al., 2016). However, a more recent study investigating the colour morph's genetic mechanisms using samples from South Africa revealed that it is instead likely to be linked to albinism pathways (Tensen et al., 2022): the red leopards tested were homozygous for a mutation on the tyrosinase‐related protein (TYRP1) gene, also referred to as the *brown* locus (Kobayashi et al., 1998).

The strawberry phenotype has only been recorded a handful of times. A 1992 study collated records of white or light‐coloured Indian leopards (*P. p. fusca*), finding five records with photographic or physical evidence (skins and trophies) between c. 1905 and 1965 (Divyabhanusinh, 1993). A female strawberry leopard was more recently photographed in Rajasthan, India in 2021 (Dsouza, 2021).

The mutation was first documented in the African leopard (*P. p. pardus*) in 2012, in a male leopard photographed in South Africa's Madikwe Game Reserve, North West Province (Dell'Amore, 2012). A 2016 study subsequently collated all known records of the colour variant in South Africa, identifying six additional cases across the Mpumalanga and North West Provinces (Pirie et al., 2016). More recently, strawberry leopards have been documented in South Africa in Thaba Tholoi Wilderness Reserve (female; Black Leopard Mountain Lodge, 2019) and Kaingo Game Reserve (male; Panthera, 2020), Limpopo Province in 2019; in Dinokeng Game Reserve, Gauteng Province, in 2020 (male; Guest Contributor, 2020); and in iSimangaliso Wetland Park, KwaZulu‐Natal Province, in late 2023 (cub of unknown sex; Ferreira, 2023). Until now, no cases have been reported on the continent outside South Africa.

## 2 MATERIALS AND METHODS

**FIGURE 1:**
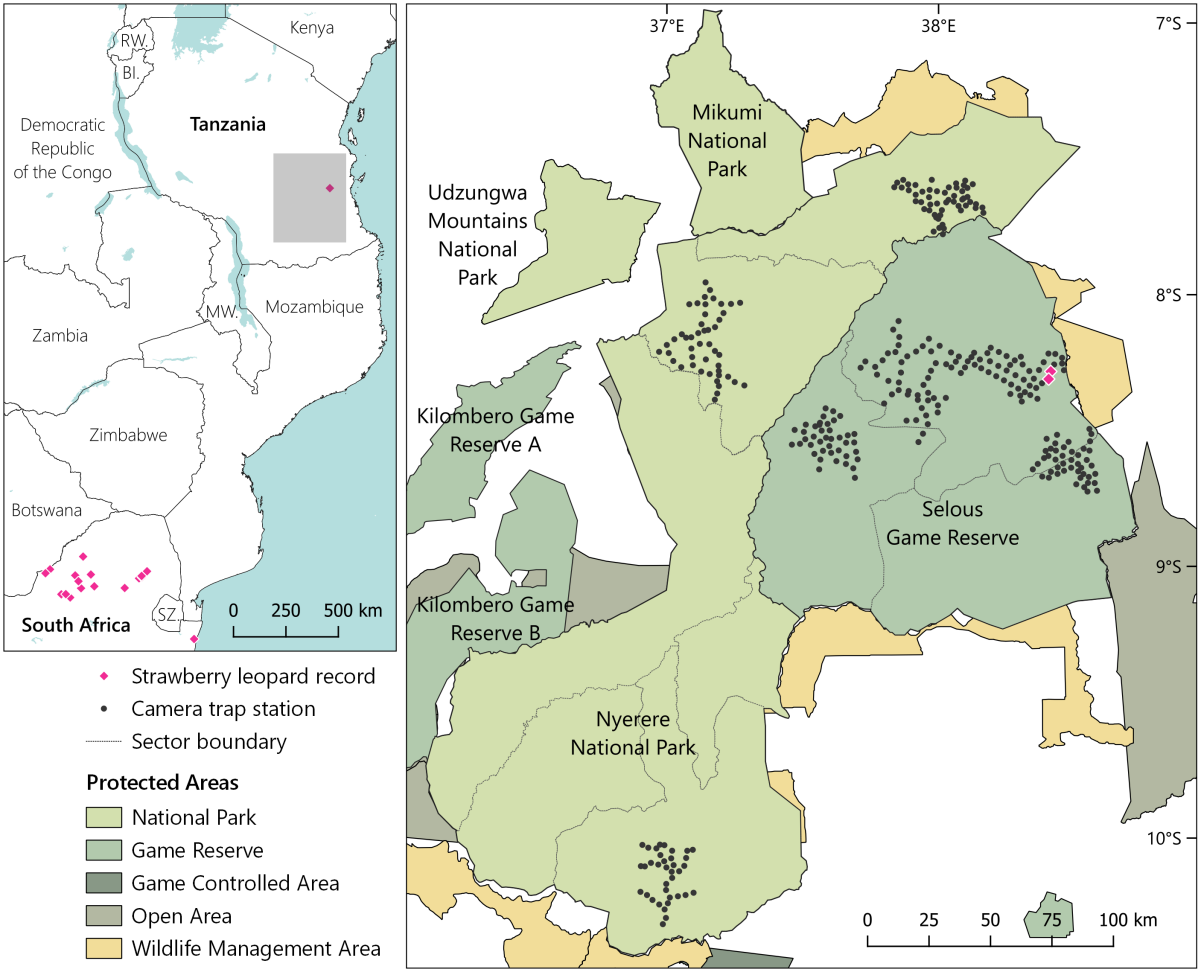
Left: location of strawberry leopard records from Africa (Pirie et al., 2016; Tensen et al., 2022). Right: map of the study area and camera trap survey grids, showing the location of the strawberry leopard in Selous GR.

The Nyerere‐Selous ecosystem is a vast conservation complex in southern Tanzania, centred on Selous Game Reserve (GR; 18,020 km^2^) and Nyerere National Park (NP; 30,893 km^2^). Between September 2020 and December 2022, we conducted camera trap surveys at seven sites in Selous GR and Nyerere NP (Figure 1), primarily to collect data suitable for spatially explicit capture‐recapture (SECR) density estimation of leopard, lion (*Panthera leo*), and spotted hyaena (*Crocuta crocuta*) as part of a large carnivore assessment (Searle, Strampelli, Haule, et al., 2023; Searle, Strampelli, Parsais, et al., 2023). Summary information for all seven camera trap surveys can be found in Table 1.

**TABLE 1:** Summary information for each of the camera trap surveys conducted in Nyerere‐Selous.

Survey ID	NMTL	NMSK	NKLC	SKPE	SKPW	SLWN	SMGN
Protected area	Nyerere NP	Nyerere NP	Nyerere NP	Selous GR	Selous GR	Selous GR	Selous GR
Management sector	Matambwe	Msolwa	Kalulu	Kingupira	Kingupira	Liwale	Miguruwe
Location within sector	Lakes	Kilombero	Central	East	West	North	Northeast
Number of stations (paired)	53	47	36	44	53	41	45
Survey area (km^2^)	527	1045	537	525	1124	507	409
Start date	4 Sep 2020	1 Aug 2022	13 Aug 2022	2 Jul 2021	14 Nov 2021	19 Jul 2021	6 Aug 2021
End date	6 Dec 2020	23 Nov 2022	17 Dec 2022	22 Nov 2021	21 Feb 2022	24 Oct 2021	24 Nov 2021
Survey duration (days)	93	114	126	143	99	97	94
Trap nights	4350	5069	4007	6166	4987	3902	3853

We used two models of motion‐activated cameras with xenon white flash (Cuddeback Professional Colour Model 1347 & X‐Change Colour Model 1279, Non Typical Inc., Wisconsin, USA). All stations were paired, consisting of one camera on each side of the road, facing the road at a 90 degree angle. Each survey comprised 36–53 paired stations covering an area of 409–1124 km^2^, and remained active for 3–5 months (Table 1). Leopards were identified from camera trap photos based on their unique spot patterns.

## 3 RESULTS

Across the seven study sites, we deployed 319 paired camera trap stations, covering a combined area of 4600 km^2^ (calculated by summing the area of the minimum convex polygon around each grid). These stations were active for a total of 32,334 camera trap nights, yielding 4028 images of leopard.

**FIGURE 2:**
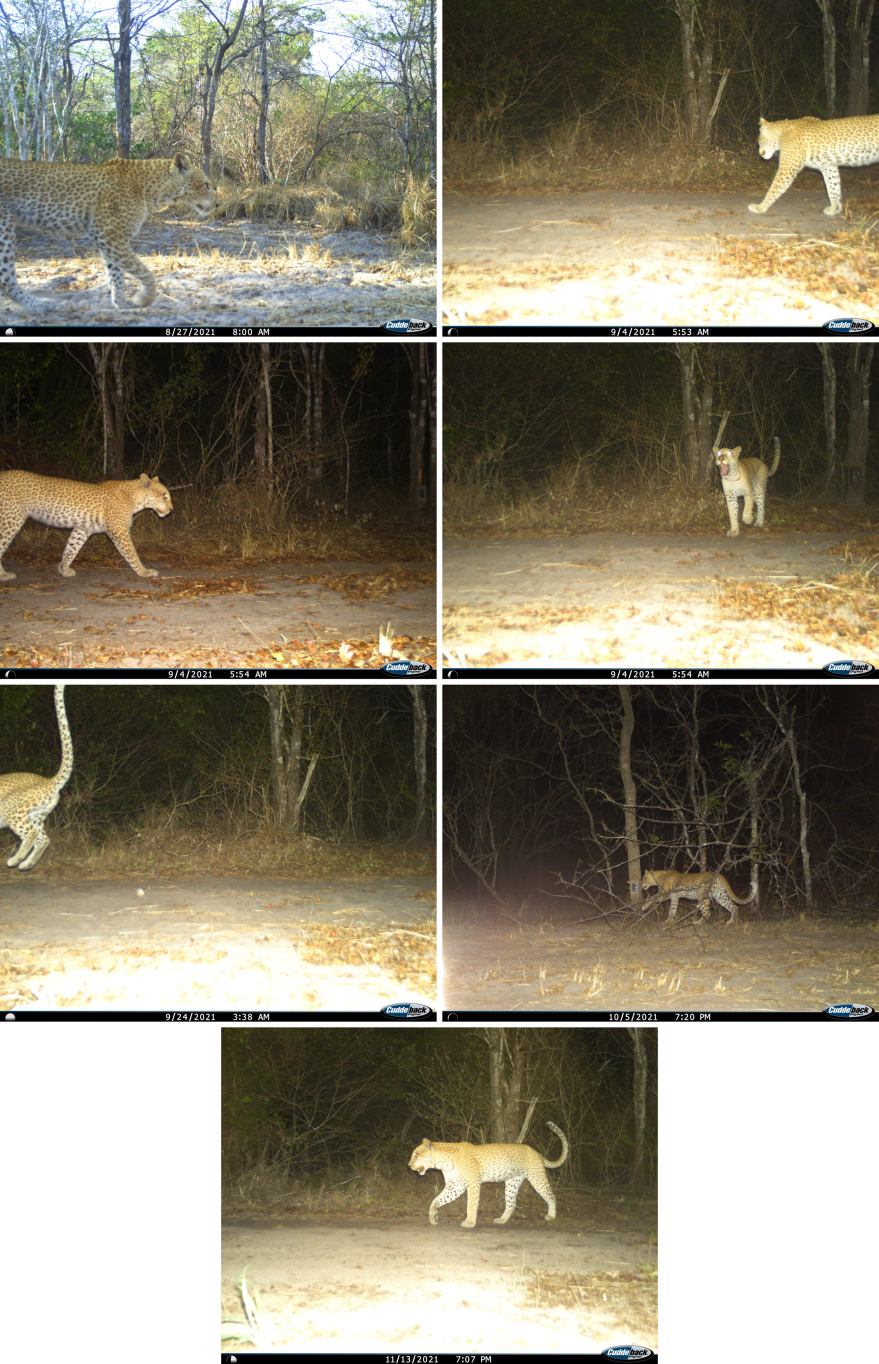
All seven photos of the strawberry leopard from five capture events in the Kingupira sector of Selous GR.

We identified 373 unique individuals, including 134 adult females, 128 adult males, 99 adults of unknown sex, and 12 cubs. Among these individuals, we documented one female leopard in the Kingupira sector of Selous GR with strawberry colouration, photographed on five occasions at two different camera trap stations between August and November 2021 (Figure 1; see Table 2 for details of all capture events and Figure 2 for all photos).

**TABLE 2:** Summary information for the five capture events of the strawberry leopard photographed in the Kingupira sector of Selous GR.

Capture event	Date	Time	Station	X_UTM37S	Y_UTM37S	X_WGS84	Y_WGS84	Elevation (m)	Images
1	27/08/2021	08:00	38	434,342	9,081,341	38.4037	−8.3104	126	1
2	04/09/2021	05:53	23	435,198	9,084,417	38.4116	−8.2826	110	3
3	24/09/2021	03:38	23	435,198	9,084,417	38.4116	−8.2826	110	1
4	05/10/2021	19:20	38	434,342	9,081,341	38.4037	−8.3104	126	1
5	13/11/2021	19:07	23	435,198	9,084,417	38.4116	−8.2826	110	1

**FIGURE 3:**
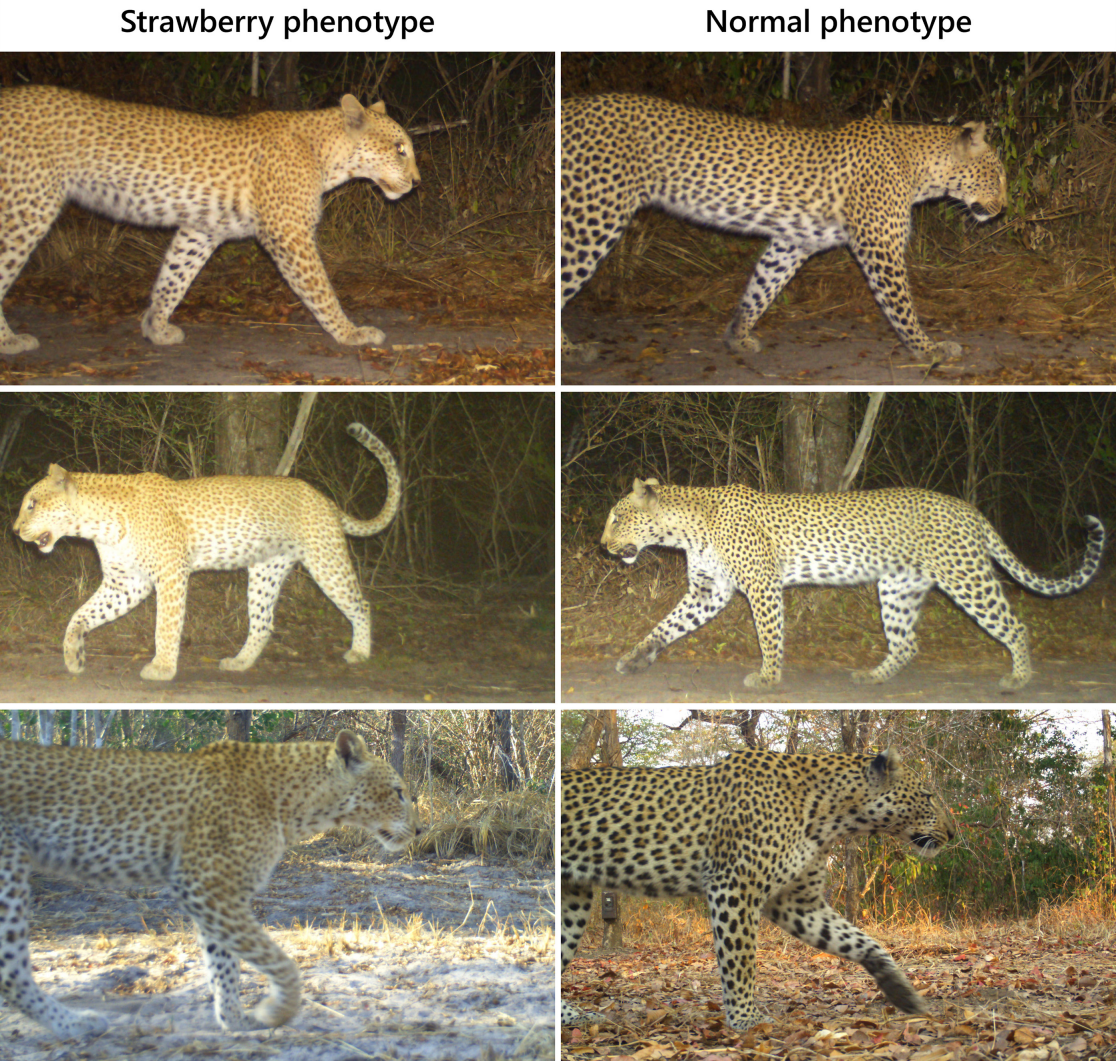
Photos of the strawberry leopard in Selous GR (left) next to a normal phenotype leopard (right), for comparison. Top: captured at the same camera, walking right in the same position; middle: captured at the same camera, walking left in the same position; and bottom: daytime photos from different cameras.

**FIGURE 4:**
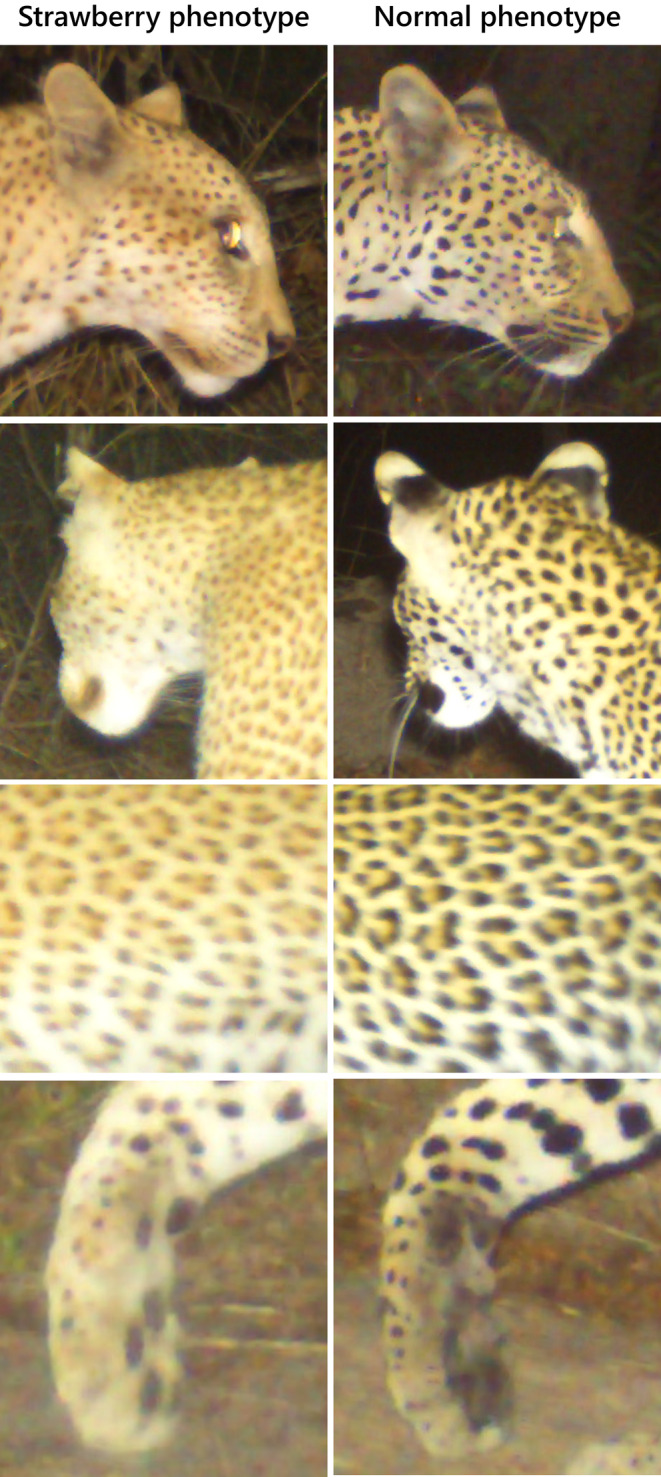
Close‐up photos of features of the strawberry leopard (left) next to those of normal phenotype female leopards (right). Top: right side of the face; upper middle: back of the head and ears; lower middle: left flank; and bottom: underside of the paw. Note the lack of black pigmentation around the mouth, on the backs of the ears, and in the fur around the paw pads.

A comparison of the strawberry leopard's colouration with standard phenotype leopards photographed at a comparable distance during both the night and day highlights her lighter colouration (Figure 3). Closer inspection also reveals a lack of black pigmentation around the mouth, on the backs of the ears, and in the fur on the underside of the paw (Figure 4).

No strawberry leopards were documented during similar camera trap surveys conducted from 2018 to 2019 in the Ruaha‐Rungwa landscape, also in southern Tanzania, which together yielded 95 identified individuals (Searle et al., 2021).

## 4 DISCUSSION

The Selous strawberry leopard's brown markings are not as light as some other documented cases (Tensen et al., 2022), and the flesh of her paw pads appears to be dark (Figure 3). It is therefore possible that this leopard is in fact part of a natural colour gradient, and not a true strawberry leopard. Ideally this would be confirmed by collecting genetic samples and testing these for the linked gene variant (TYRP1 exon 2). However, we suspect that she is heterozygous in one of the red leopard alleles, as suggested for the male recorded in Dinokeng GR, South Africa in 2020, which also exhibited darker brown spots (Tensen et al., 2022).

This is the first documented record of a suspected strawberry leopard on the African continent outside South Africa, at a distance of approximately 2000 km from the closest existing record (Figure 1). It is possible that this is not the first occurrence of a strawberry leopard in Nyerere‐Selous, but that the mutation had simply not been detected until now due to a lack of surveillance.

In addition to being rare within Africa, the colouration was rare within our dataset: the strawberry colouration female represented 0.3% of individuals identified in Nyerere‐Selous, dropping to 0.2% when combined with data from the nearby Ruaha‐Rungwa landscape. This is a much lower frequency than the ~3.6% recently estimated in North West and Mpumalanga Provinces of South Africa (Tensen et al., 2022).

The presence of a strawberry leopard in Selous could be the result of a stepping‐stone dispersal from northern South Africa. However, 2000 km is a considerable distance – male leopards in a South African population were found to disperse 11 km on average (Fattebert et al., 2015), although one dispersal event of 350 km has been recorded (Fattebert et al., 2013) – particularly as the phenotype is thought to occur in South Africa as a result of males dispersing shorter than usual distances (Tensen et al., 2022; Tensen & Fischer, 2024). Dispersers would also have had to successfully navigate through extensive areas of non‐habitat, as well as major rivers (particularly the Zambezi and Ruvuma Rivers). Furthermore, no other records of this phenotype are available from between these two areas.

It is possible that the strawberry colouration offers a selective benefit for leopards in the landscape, like the potential higher hunting success of white morphs of the black bear (*Ursus americanus kermodei*; Klinka & Reimchen, 2009) and possible advantage of the servaline serval's smaller spots in forested areas (*Leptailurus serval*; Bantlin & Evers, 2023). However, if this were the case, we would expect the morph to be more common within the Selous leopard population than observed (Tensen & Fischer, 2024).

Alternatively, the strawberry phenotype could have arisen in Selous as a result of heterozygote deficiency due to inbreeding (Tensen & Fischer, 2024), as the leopard population is subject to trophy hunting (Searle, Strampelli, Parsais, et al., 2023). This is the mechanism believed to be behind the phenotype's proliferation in northern South Africa, where a high offtake of adult males has meant that young males do not need to disperse far from their mothers, which increases inbreeding and thus the chance of rare recessive alleles being expressed (Pirie et al., 2016; Tensen et al., 2022). This could therefore be evidence of disruptive population dynamics among leopards in the Nyerere‐Selous ecosystem, and warrants further investigation.

We recommend that those collecting camera trap data in areas with leopards, particularly between northern South Africa and southern Tanzania, look out for individuals with strawberry colouration to understand if the variant is more widespread than currently thought. Where individuals with this colouration are detected, efforts should be made to collect and test genetic samples to establish whether these individuals are true strawberry leopards.

## AUTHOR CONTRIBUTIONS

**Charlotte E. Searle:** Conceptualization (equal); data curation (equal); funding acquisition (equal); project administration (equal); writing – original draft (lead); writing – review and editing (equal). **Paolo Strampelli:** Conceptualization (equal); data curation (equal); funding acquisition (equal); project administration (equal); writing – original draft (supporting); writing – review and editing (equal). **Singira N. Parsais:** Data curation (equal); writing – review and editing (equal). **Leonard J. Haule:** Data curation (equal); writing – review and editing (equal). **Kandey K. Olesyapa:** Data curation (equal); writing – review and editing (equal). **Nasri D. Salum:** Data curation (equal); writing – review and editing (equal). **Germanus Hape:** Data curation (equal); writing – review and editing (equal). **Manase Elisa:** Data curation (equal); writing – review and editing (equal). **Daniel Mathayo:** Data curation (equal); writing – review and editing (equal). **Joseph Kaduma:** Data curation (equal); writing – review and editing (equal). **Neema Malulu:** Data curation (equal); writing – review and editing (equal). **Nyasatu Mkaka:** Data curation (equal); writing – review and editing (equal). **Justine Robert:** Data curation (equal); writing – review and editing (equal). **Dennis Ikanda:** Data curation (equal); writing – review and editing (equal). **Samuel Mtoka:** Data curation (equal); writing – review and editing (equal). **Kathryn Doody:** Conceptualization (equal); funding acquisition (equal); project administration (equal); writing – review and editing (equal). **Alex L. Lobora:** Conceptualization (equal); project administration (equal); writing – review and editing (equal). **Amy J. Dickman:** Conceptualization (equal); funding acquisition (equal); project administration (equal); writing – review and editing (equal).

## CONFLICT OF INTEREST STATEMENT

The authors declare no conflicts of interest.

## Data Availability

All capture data and images of the strawberry leopard can be found in Table 2.
